# Health Care Utilization and Outcomes Associated with Accidental Poisonous Mushroom Ingestions — United States, 2016–2018

**DOI:** 10.15585/mmwr.mm7010a1

**Published:** 2021-03-12

**Authors:** Jeremy A.W. Gold, Emily Kiernan, Michael Yeh, Brendan R. Jackson, Kaitlin Benedict

**Affiliations:** ^1^Epidemic Intelligence Service, CDC; ^2^Division of Foodborne, Waterborne, and Environmental Diseases, National Center for Emerging and Zoonotic Infectious Diseases, CDC; ^3^Agency for Toxic Substances and Disease Registry, Atlanta, Georgia; ^4^Department of Emergency Medicine, Emory University School of Medicine, Atlanta, Georgia.

Accidental consumption of poisonous mushrooms can result in serious illness and death (*1*). Reports of severe poisonings from consumption of foraged mushrooms for food or hallucinogenic purposes increased during 1999–2016 (*2*), and approximately 7,500 poisonous mushroom ingestions were reported annually to poison control centers across the United States (*1*). To estimate the frequency of emergency department (ED) visits, hospitalizations, and severe adverse outcomes associated with accidental poisonous mushroom ingestion in the United States, CDC analyzed 2016 data from the Healthcare Cost and Utilization Project’s* Nationwide Emergency Department Sample (HCUP-NEDS) and National Inpatient Sample (HCUP-NIS) databases as well as 2016–2018 data from three IBM MarketScan sources: Commercial Claims and Encounters (CCAE), Medicare Supplemental and Coordination of Benefits (Medicare), and Multi-State Medicaid databases. During 2016, 1,328 (standard error [SE] = 100) ED visits and 100 (SE = 22) hospitalizations (HCUP data) were associated with accidental poisonous mushroom ingestion. Among 556 patients with a diagnosis of accidental poisonous mushroom ingestion, 48 (8.6%) patients experienced a serious adverse outcome during 2016–2018 (MarketScan data). Serious adverse outcomes were more common among Medicaid-insured patients than among patients with commercial insurance or Medicare (11.5% versus 6.7%, p = 0.049). Because most mushroom poisonings are preventable, wild mushrooms should not be consumed unless they are identified by an expert; increased public health messaging about the potential dangers of mushroom poisoning is needed.

CDC analyzed 2016 data from HCUP-NEDS and HCUP-NIS, the largest publicly available databases for all-payer ED visits and hospitalizations, respectively. (At the time the analysis was performed, 2016 was the most recent year of data available). HCUP data were accessed through HCUPnet, a free, web-based platform (*3*). These databases produce national estimates of patient health care use and charges by insurance payer status, U.S. Census region, and urban-rural status of patient residence,^†^ without deduplication of multiple visits per patient. To produce patient-level analyses, this study also analyzed IBM MarketScan data, which were accessed through Treatment Pathways,^§^ a web-based platform with data from patients whose health insurance plan or employer contributes prescription drug data to MarketScan. The 2016–2018 MarketScan CCAE and Medicare claims data included outpatient visits and hospitalizations for approximately 34,900,000 patients throughout the United States; the Medicare data set included retirees with employer-sponsored Medicare supplemental insurance. The 2016–2018 MarketScan Medicaid data sets included similar information for approximately 11,000,000 Medicaid patients from across several states. Data on race and ethnicity were available only in the Medicaid data sets. None of the databases (HCUP or MarketScan) included data on deaths from ingestion of poisonous mushrooms. *International Classification of Diseases, Tenth Revision, Clinical Modification* (ICD-10-CM) codes^¶^were used to identify diagnoses for accidental poisonous mushroom ingestion and associated clinical signs, symptoms, diagnoses, hallucinogenic drug use, and serious adverse outcomes (defined as potentially dangerous cardiac arrhythmia, renal failure, liver failure, rhabdomyolysis, seizure, or respiratory failure) occurring within 72 hours of ingestion. Because most poisonous mushroom ingestions reported to U.S. poison centers occur in young children (*1*), patients from MarketScan were categorized as being ≤5 years or >5 years. HCUPnet provides fixed age categorizations (<1, 1–17, 18–44, 45–64, 65–84, and ≥85 years) for patients in its databases. This study used data from the MarketScan databases to compare differences in demographic characteristics, diagnoses, and adverse outcomes between these age groups and between insurance types (Medicaid versus CCAE or Medicare) using chi-square or Fisher’s exact tests for proportions. Analyses were performed using Epi Info (version 7.2.3.1; CDC); p-values <0.05 were considered statistically significant. This activity was reviewed by CDC and conducted consistent with applicable federal law and CDC policy.^**^

In the 2016 HCUP databases, an estimated 1,328 (SE = 100) ED visits associated with accidental poisonous mushroom ingestions occurred in the United States (Table 1). Among ED visits, the most common insurance types were private (567 [42.7%], SE = 69), Medicaid (451 [34.0%], SE = 53), and none (187 [14.1%], SE = 30). By region, most ED visits occurred in the West (495 [37.3%], SE = 67), followed by the South (372 [28%], SE = 43), the Midwest (246 [18.5%], SE = 44), and the North (214 [16.1%], SE = 42). The most common residence settings were medium and small metropolitan (497 [37.4%], SE = 58) and suburban (335 [25.2%], SE = 55) areas. An estimated 100 (SE = 22) hospitalizations associated with accidental mushroom ingestions occurred in 2016. The estimated mean length of stay for hospitalized patients was 2.4 days (SE = 0.4). The estimated mean annual cost per hospitalization was $7,626 (SE = 1,407), and aggregate national hospitalization costs were $762,574 (SE = 220,216).

**TABLE 1:** Emergency department visits (N = 1,328) associated with ingestion of poisonous mushrooms* — United States, 2016

Characteristic	No. (%)	SE
**Age group, yrs**		
<1	—^†^	—
1–17	548 (41.3)	61
18–44	511 (38.5)	52
45–64	180 (13.6)	30
≥65	—	—
**Male**	832 (62.7)	73
**Payer**		
Private insurance	567 (42.7)	69
Medicaid	451 (34.0)	53
Uninsured	187 (14.1)	30
Medicare	—	—
Other	—	—
**U.S. Census region** ^§^		
West	495 (37.3)	67
South	372 (28.0)	43
Midwest	246 (18.5)	44
Northeast	214 (16.1)	42
**Patient residence** ^¶^		
Large central metropolitan	267 (20.1)	43
Large fringe metropolitan (suburbs)	335 (25.2)	55
Medium and small metropolitan	497 (37.4)	58
Micropolitan and noncore (rural)	213 (16.0)	41
Missing	—	—

**Abbreviation:** SE = standard error.

* Healthcare Cost and Utilization Project (HCUP) data include weighted national estimates from HCUP Nationwide Emergency Department Sample. Poisonous mushroom ingestion-associated emergency department visits were identified using *International Classification of Diseases, Tenth Revision* (ICD-10) code T62.0X1.

^†^ Dashes indicate that statistics based on estimates with a relative SE (standard error/weighted estimate) >0.30 or with SE error = 0 are suppressed.

^§^ *Northeast*: Connecticut, Maine, Massachusetts, New Hampshire, New Jersey, New York, Pennsylvania, Rhode Island, and Vermont; *Midwest*: Illinois, Indiana, Iowa, Kansas, Michigan, Minnesota, Missouri, Nebraska, North Dakota, Ohio, South Dakota, and Wisconsin; *South*: Alabama, Arkansas, Delaware, District of Columbia, Florida, Georgia, Kentucky, Louisiana, Maryland, Mississippi, North Carolina, Oklahoma, South Carolina, Tennessee, Texas, Virginia, and West Virginia; *West*: Alaska, Arizona, California, Colorado, Hawaii, Idaho, Montana, Nevada, New Mexico, Oregon, Utah, Washington, and Wyoming.

^¶ ^In HCUP data, patient residence is classified using the National Center for Health Statistics Urban-Rural Classification Scheme for Counties (https://www.cdc.gov/nchs/data_access/urban_rural.htm).

Among 556 patients with diagnosed poisonous mushroom ingestion in the combined 2016–2018 MarketScan databases, 329 (59.2%) were from the CCAE and Medicare data sets, and 227 (40.8%) were from the Medicaid data set (Table 2). In the Medicaid data set, 61.7% of patients were White, 16.3% of patients were Black, and 22.0% of patients were classified as other or missing race. Overall, 144 (25.9%) patients were aged ≤5 years, 412 (74.1%) were aged >5 years, and 311 (55.9%) were male. The most common care setting for diagnosis of poisonous mushroom ingestion was an ED (79.5%), and diagnosis was more likely during summer (38.5%). Patients aged >5 years were hospitalized more often (p = 0.010). Compared with patients aged ≤5 years, older patients were more likely to have any documented symptoms or clinical findings associated with mushroom poisoning (68.9% versus 28.5%, p<0.001) and any documented laboratory abnormalities (14.1% versus 4.9%, p = 0.003). The most common associated symptoms were gastrointestinal (36.0%), neurologic/behavioral (18.3%), cardiac (16.5%), and respiratory (4.0%). Hallucinogenic drug use was documented in 43 (10.4%) patients aged >5 years.

**TABLE 2:** Characteristics of patients with diagnosis of accidental poisonous mushroom ingestion, by age group — United States, 2016–2018*

Characteristic	MarketScan Commercial Claims and Encounters and Medicare				MarketScan Medicaid				Combined MarketScan (commercial and Medicaid)			
	No. (%)			p-value^†^	No. (%)			p-value^†^	No. (%)			p-value^†^
	All patients (N = 329)	Age group			All patients (N = 227)	Age group			All patients (N = 556)	Age group		
		≤5 yrs (n = 66)	>5 yrs (n = 263)			≤5 yrs (n = 78)	>5 yrs (n = 149)			≤5 yrs (n = 144)	>5 yrs (n = 412)	
**Male**	179 (54.4)	34 (51.5)	145 (55.1)	0.60	132 (58.1)	41 (52.6)	91 (61.1)	0.22	311 (55.9)	75 (52.1)	236 (57.3)	0.28
**Season of diagnosis**												
Winter	48 (14.6)	5 (7.6)	43 (16.3)	0.31	20 (8.8)	7 (9.0)	13 (8.7)	0.94	68 (12.2)	12 (8.3)	56 (13.6)	0.42
Spring	74 (22.5)	16 (24.2)	58 (22.1)		45 (19.8)	17 (21.8)	28 (18.8)		119 (21.4)	33 (22.9)	86 (20.9)	
Summer	121 (36.8)	28 (42.4)	93 (35.4)		93 (41.0)	30 (38.5)	63 (42.3)		214 (38.5)	58 (40.3)	156 (37.9)	
Autumn	86 (26.1)	17 (25.8)	69 (26.2)		69 (30.4)	24 (30.8)	45 (30.2)		155 (27.9)	41 (28.5)	114 (27.7)	
**Care setting** ^§^												
Inpatient	16 (4.9)	—^¶^	16 (6.1)	0.049	21 (9.3)	—	18 (12.1)	0.053	37 (6.7)	—	34 (8.3)	0.010
Emergency department	246 (74.8)	60 (90.9)	186 (70.7)	0.001	196 (86.3)	72 (92.3)	124 (83.2)	0.058	442 (79.5)	132 (91.7)	310 (75.2)	<0.001
Outpatient office	151 (45.9)	12 (18.2)	139 (52.9)	<0.001	84 (37.0)	17 (21.8)	67 (45.0)	0.001	235 (42.3)	29 (20.1)	206 (50.0)	<0.001
**Symptoms and clinical findings**	194 (59.0)	11 (16.7)	183 (69.6)	<0.001	131 (57.7)	30 (38.5)	101 (67.8)	<0.001	325 (58.4)	41 (28.5)	284 (68.9)	<0.001
Gastrointestinal	103 (31.3)	7 (10.6)	96 (36.5)	—	97 (42.7)	27 (34.6)	70 (47.0)	—	200 (36.0)	34 (23.6)	166 (40.3)	<0.001
Neurologic/Behavioral	65 (19.8)	—	63 (24.0)	—	37 (16.3)	5 (6.4)	32 (21.5)	—	102 (18.3)	7 (4.9)	95 (23.1)	<0.001
Cardiac	51 (15.5)	—	49 (18.6)	—	41 (18.1)	5 (6.4)	36 (24.2)	—	92 (16.5)	7 (4.9)	85 (20.6)	<0.001
Respiratory	10 (3.0)	—	9 (3.4)	—	12 (5.3)	—	12 (8.1)	—	22 (4.0)	—	21 (5.1)	0.022
Allergic	10 (3.0)	—	9 (3.4)	—	—	—	—	—	13 (2.3)	—	12 (2.9)	0.20
Any laboratory abnormalities	32 (9.7)	—	31 (11.8)	—	33 (14.5)	6 (7.7)	27 (18.1)	0.034	65 (11.7)	7 (4.9)	58 (14.1)	0.003
Hallucinogenic drug use	27 (8.2)	—	27 (10.3)	0.004	16 (7.0)	—	16 (10.7)	0.002	43 (7.7)	—	43 (10.4)	<0.001
**Serious adverse outcomes**	22 (6.7)	—	22 (8.4)	0.011	26 (11.5)	—	22 (14.8)	0.030	48 (8.6)	—	44 (10.7)	0.003

* IBM MarketScan Commercial Claims and Encounters/Medicare Supplemental and Coordination of Benefits, IBM MarketScan Multi-State Medicaid, and combined MarketScan databases during 2016–2018 were analyzed for patient demographics, health care setting, clinical features, and outcomes.

† P-values for IBM MarketScan Commercial Claims and Encounters/Medicare, IBM MarketScan Multi-State Medicaid, and combined databases were calculated using Fisher’s exact tests or chi-square tests for proportions comparing patient age groups.

^§^ Patients might have received care in more than one setting.

^¶^ Dashes indicate the number of patients was less than five, and values are suppressed.

During 2016–2018, serious adverse outcomes occurred in 48 (8.6%) patients overall and were more common in patients aged >5 years than in patients aged ≤5 years (p = 0.003). The most common serious adverse outcome was cardiac arrythmia (2.7%), followed by acute renal failure (2.2%), liver failure (1.8%), rhabdomyolysis (1.4%), and seizure (1.4%). Serious adverse outcomes were more common in Medicaid-insured patients than among patients with commercial insurance or Medicare (11.5% versus 6.7%, p = 0.049).

## Discussion

This study, which analyzed administrative data sets, found that 1,328 accidental poisonous mushroom ingestions were treated in EDs during 2016, and during 2016–2018 serious adverse outcomes occurred in 8.6% (48 of 556) of patients who sought care for accidental poisonous mushroom ingestions. Serious adverse outcomes were more common in patients with Medicaid than in patients with commercial or Medicare insurance, suggesting that severe consequences of poisonous mushroom ingestions might be more common among patients with lower socioeconomic status. A small proportion of patients aged >5 years (10%) received a diagnosis of hallucinogenic drug ingestion, suggesting that most accidental mushroom poisonings occurred because of consumption of foraged mushrooms for food rather than ingestion for hallucinogenic purposes.

Adverse outcomes from poisonous mushroom ingestions might occur because amateur mushroom foragers might not distinguish poisonous from nonpoisonous species (*2*). Recent immigrants are also at risk for mushroom poisonings because they might mistake poisonous mushrooms for nontoxic varieties found in other countries (*4*). Accidental mushroom poisoning diagnoses were more common in the summer and most frequently occurred in the western United States; this might reflect regional differences in the popularity of recreational mushroom foraging or the fact that *Amanita smithiana*, a potentially deadly and easily misidentified mushroom species that causes gastrointestinal symptoms followed by acute renal failure, is more common in this region (*5*). The public should be aware that poisonous mushrooms might resemble nonpoisonous mushrooms, cooking mushrooms does not remove or inactivate toxins, and that wild mushrooms should never be consumed unless they are identified by an expert (*6*).

This analysis demonstrates the potential for serious adverse outcomes in young children, although they occurred less frequently than in older persons. Young children might take small, exploratory bites of mushrooms during outdoor play; older persons might be more likely to consume larger quantities of mushrooms as food or to achieve a hallucinogenic effect (*7*). Studies have documented serious adverse outcomes related to poisonous mushroom ingestions in young children, including liver failure and permanent neurologic impairment (*8*,*9*). The public should be aware that children should be supervised when playing outside in areas where mushrooms might grow, and children should not be fed wild mushrooms unless the mushrooms are identified by experts.

Previous studies have found that patients with Medicaid are less likely to use poison control centers in general, potentially resulting in higher rates of unnecessary ED visits, preventable adverse outcomes, and costly hospitalizations from accidental poisonings (*10*). The reasons for more frequent adverse outcomes from poisonous mushroom ingestions in Medicaid-insured patients merits further study. Medicaid patients might benefit from targeted public health messaging regarding mushroom poisonings.

The findings in this report are subject to at least six limitations. First, HCUP data do not allow patient-level analyses, and the MarketScan databases represent a large convenience sample. Second, using both types of data sources (HCUP and MarketScan) permitted examination of the national prevalence of mushroom poisoning and analysis of patient characteristics; however, these administrative data sets are subject to inconsistent ICD-10-CM coding and misclassification. Third, because race and ethnicity data were unavailable for patients with commercial insurance in the MarketScan database, outcome comparison by race and ethnicity was not possible for most patients in the analysis. Fourth, the broad age groups of patients compared in the MarketScan databases did not distinguish between older children and adults; to address this limitation, analyses were repeated using more discrete age categorizations, and the frequency of serious adverse events was lower in children aged ≤5 years compared with all other age groups. Fifth, information about deaths was not available. Finally, because many poisonous mushroom ingestions are likely not reported, this report might underestimate the actual public health effects of poisonous mushroom ingestion.

Although mushroom poisoning is relatively uncommon, it can result in severe illness. Because most illnesses from poisonous mushroom ingestion are preventable, increased public awareness about the potential dangers of mushroom poisoning is needed. Given the potential severity and preventable nature of most poisonous mushroom ingestions, wild mushrooms should not be consumed unless identified by an expert and continued public health messaging about this topic is warranted.

**Summary**

**What is already known about this topic?**

Poisonous mushroom ingestions can result in serious illness and death. The national prevalence of health care use associated with accidental poisonous mushroom ingestion is unknown.

**What is added by this report?**

During 2016, an estimated 1,328 emergency department visits and 100 hospitalizations were associated with accidental poisonous mushroom ingestion. During 2016–2018, 8.6% (48 of 556) of patients who sought care for poisonous mushroom ingestions had a serious adverse outcome. Serious adverse outcomes were more common in Medicaid-insured patients than commercially insured patients.

**What are the implications for public health practice?**

Wild mushrooms should not be consumed unless identified by an expert; continued public health messaging about the potential dangers of poisonous mushroom ingestions is needed.

## Footnotes

*
https://www.hcup-us.ahrq.gov/

† In Healthcare Cost and Utilization Project data, patient residence is classified using the National Center for Health Statistics Urban-Rural Classification Scheme for Counties (https://www.cdc.gov/nchs/data_access/urban_rural.htm).

§
https://www.ibm.com/products/marketscan-treatment-pathways

¶ Toxic effect of ingested mushrooms, accidental (T62.0X1); gastrointestinal symptoms and diagnoses (K29.70, K52.1, K52.8, K52.9, R10–R11, and R19.7); neurologic or behavioral symptoms and diagnoses (G40.89, G47.00, G93.40, R20.2, R40, R41.0, R41.82, R41.89, R42, R44, R46.2, R53.1, R56.9, and Z74.3); cardiac symptoms and diagnoses (I30.9, I45.10, I45.4, I47.2, I49.9, I50.9, R00.0–R00.2, R00.8, R00.9, R07.9, R55, and R94.31); respiratory symptoms and diagnoses (J96, R05, R06.00, R06.02, and R06.89); allergic diagnoses (T78.00X, T78.2XX, and T78.3XX); laboratory abnormalities (D64.9, D72.819, D72.82, E87.5, E87.6, M62.82, N17.9, R73.9, R74.0, R74.8, R79.89, and R94.5); hallucinogenic drug use (F12.1, F16.1, T40.8X, and T43.64); arrythmia (I45.10, I45.4, I47.2, I49.9, R00.1, and R00.9); acute renal failure (N17 and N19); liver failure (K71, K72.00, K72.01, K72.90, and K72.91); rhabdomyolysis (M62.82); seizure (G40.89, R56.9); and respiratory failure (J96.01, J96.90).
